# Evaluation of Methods for Sizing and Counting of Ultrasound Contrast Agents

**DOI:** 10.1016/j.ultrasmedbio.2012.01.012

**Published:** 2012-05

**Authors:** Charles A. Sennoga, James S.M. Yeh, Julia Alter, Eleanor Stride, Petros Nihoyannopoulos, John M. Seddon, Dorian O. Haskard, Joseph V. Hajnal, Meng-Xing Tang, Robert J. Eckersley

**Affiliations:** ∗ Imaging Sciences Department, Imperial College London, Hammersmith Campus, London, United Kingdom; † Department of Bioengineering, Imperial College London, South Kensington Campus, London, United Kingdom; ‡ Department of Chemistry, Imperial College London, South Kensington Campus, London, United Kingdom; § National Heart and Lung Institute, Imperial College London, Hammersmith Campus, London, United Kingdom; ‖ Department of Mechanical Engineering, University College London, Torrington Place, London, United Kingdom

**Keywords:** Microbubble number count, Mean size diameter, Size distribution, Electro-impedance volumetric zone sensing, Optical microscopy, Laser diffraction, Intra-method variation, SonoVue™

## Abstract

A precise, accurate and well documented method for the sizing and counting of microbubbles is essential for all aspects of quantitative microbubble-enhanced ultrasound imaging. The efficacy of (a) electro-impedance volumetric zone sensing (ES) also called a Coulter counter/multisizer; (b) optical microscopy (OM); and (c) laser diffraction (LD), for the sizing and counting of microbubbles was assessed. Microspheres with certified mean diameter and number concentration were used to assess sizing and counting reproducibility (precision) and reliability (accuracy) of ES, OM and LD. SonoVue™ was repeatedly (n = 3) sized and counted to validate ES, OM and LD sizing and counting efficacy. Statistical analyses of intra-method variability for the SonoVue™ mean diameter showed that the best microbubble sizing reproducibility was obtained using OM with a mean diameter sizing variability of 1.1%, compared with a variability of 4.3% for ES and 7.1% for LD. The best microbubble counting reproducibility was obtained using ES with a number concentration variability of 8.3%, compared with a variability of 22.4% for OM and 32% for LD. This study showed that no method is fully suited to both sizing and counting of microbubbles.

## Introduction and Literature

In recent years, microbubble ultrasound contrast agents have become increasingly important in medicine (Blomley et al. 2001). In the biomedical sciences, microbubbles have been employed as agents for metabolic gas delivery (Bisazza et al. 2008; Burkard and Van Liew 1994; Swanson et al. 2010), as mediators of drug and gene delivery (Lu et al. 2003; Porter et al. 1996; Shohet et al. 2000; Tachibana et al. 1999), as tools for molecular imaging (Klibanov 1999; Lindner 2004), in addition to their conventional use in image enhancement during ultrasound diagnostic examinations (Becher and Burns 2000; Dawson 1999). The ultrasonic backscatter and, thus, utility of these ultrasound contrast agents depends not only on their number concentrations but also on their size distribution (Gorce et al. 2000) as well as acoustic pressures used (Becher and Burns 2000).

A well-documented, accurate and precise method for the counting and sizing of microbubble ultrasound contrast agents is essential for all aspects of quantitative microbubble-enhanced ultrasound imaging. The traditional method for determining microbubble size and/or number concentration has been a manual counting chamber (haemocytometer) and a light microscope coupled with image analysis, hereafter optical microscopy (OM) (Alter et al. 2009; Lentacker et al. 2006). A variety of automated technologies including electro-impedance volumetric sensing (ES), also referred to as a Coulter counter or Coulter multisizer *e.g.,* (Chapman et al. 2005; Feshitan et al. 2009; Rychak et al. 2006; Schumann et al. 2002; Weller et al. 2005) and laser diffraction (LD) *e.g.*, (Farook et al. 2007; Lentacker et al. 2006; Moran et al. 2006) have been employed in characterising microbubble size distribution and/or concentration. Although ES, OM and LD have been employed for many years to characterise microbubble dispersions, we know of no study that validates the effectiveness of these disparate microbubble sizing and counting methods. A validation of the commonly used methods is needed for a variety of reasons. For example, current generation ES analysers have a narrow size detection range of 0.6–30 μm in diameter for the Coulter multisizer II used in this study compared with a broad size detection range of 0.02–2000 μm for Malvern’s Mastersizer 2000 employing low-angle laser light scattering combined with backscattering. This difference in detection range, likely result in under-representation of the very fine particle sizes by the ES method. Modern LD instruments utilise a large number of detectors, which used together with back scattering, the combination of different wavelength, white light scattering and improved mathematical models, not only increases the detection range but also allows for significant improvements in the quantification of the very fine particles. This has practical implications as under-representation of small bubbles will likely lead to microbubble self-attenuation, which is a confounding factor in microbubble-ultrasound imaging (Tang et al. 2008). Perhaps as a consequence of its purported sizing superiority, LD-based size characterisation of microbubbles has been employed in characterising various microbubble agents (see for example Farook et al. 2007; Lentacker et al. 2006; Moran et al. 2006).

The purpose of the present article, therefore, was to assess microbubble sizing and counting efficacies of ES, OM and LD. First, instrument precision as well as sizing and counting accuracy were determined using replicate measurements of mean diameter and number concentration of microspheres with certified mean diameter and number concentration. Second, the efficacy of each method was assessed by determining the intra-method variability of the mean diameter and number concentration for the microbubble ultrasound contrast agent, SonoVue™ (Bracco SpA, Milano, Italy).

### Description of techniques

Detailed discussions of particle sizing and counting methods may be found in various publications (see *e.g.*, Syvitski 1991) and will not be repeated here. Rather, the methods evaluated in this study are briefly reviewed.

### Electro-impedance volumetric zone sensing (ES)

ES is based on the Coulter principle (Coulter 1956). In a typical set-up, test particles are dispersed in a conductive liquid and homogenized by stirring. A tube, which is sealed on one end and connected to a precision pump on the other, is immersed into the dispersion of test particles. Through a short and narrow aperture in the tube, hereafter a sensing zone, the dispersion comprising the test particles is drawn into the tube while simultaneously monitoring the electrical conductance across the sensing zone using electrodes of opposite polarity, positioned on either side of the sensing zone. Because transiting particles obstruct part of the sensing zone, the electrical conductance will decrease momentarily each time a particle transits through the aperture. Changes in electrical conductance due to particle transitions are recorded as impedance pulses. Pulses collected as a function of measurement duration are passed to a pulse-height analyzer where they are counted and scaled to produce a number- or mass-weighted particle size distribution. Particle concentration is derived from pulse counts after correcting for the dilution fraction employed. Coincident passage of two or more particles through the sensing zone is a potential source of error as this causes the pulse-height analyser to erroneously assign the combined pulse height of multiple small particles as a single particle, thereby skewing the size distribution toward the higher end of the size range. Modern instruments, including the one used in this study, overcome this limitation by employing a pulse discrimination system that corrects for this effect by rejecting distorted pulses. Microspheres with traceable sizes are needed to calibrate a useable particle size range. This allows for the registered pulses to be correlated to a specific particle size or volume. The particle size range that can be determined using a given aperture is of the order 2% to 40 % of its diameter (Allen 1990). Consequently, differently sized apertures are required to capture broad particle size distributions. The analytical range achievable for current generation instruments is of the order 0.5 to 400 μm in diameter.

### Optical microscopy (OM)

The OM method for sizing and counting of particles involves: (1) the homogenisation of the test particles by dispersion in a non-dissolving liquid; (2) identification and image capture of a representative test ensemble using a combination of bright-field microscopy and a traceable counting chamber; followed by (3) conversion of the captured images into a particle size distribution using image processing (see also Sennoga et al. 2010). Briefly, as little as 10 μL of the homogenised dispersion of the test particles is loaded, by capillary action, into a counting chamber, hereafter a haemocytometer. Images of nonoverlapping particles that are representative of the homogenised test ensemble are captured and stored for subsequent off-line processing using sizing and counting algorithms. As all microscopic techniques can in principle be used, the detectable size range is only defined by the microscope used. With careful selection of microscope objective and imager therefore, OM can achieve a broad particle size range in which the lower limit is close to the optical resolution, and the upper limit is several millimetres at low magnification. The tiny sample sizes employed, however, creates a massive sampling problem leading to questions about the statistical relevance of the data. Consequently, a large number of observations is usually required to effect better sizing and counting reproducibility. In fact, ISO document No. 13322-1 argues that because most particle size distributions are log normal, one would need a sample size of hundreds of thousands of particles for a size distribution with 95% confidence interval. Investigations into the problem by Vigneau et al. (2000) have found a much smaller sample size to be satisfactory. These authors propose satisfying this requirement by measuring particles until the data converges at some average value for a plot of observed diameter vs. sample size. For poly-disperse particles, errors can occur due to the inadequate number of particles counted especially for particles with diameters near the toes of the distribution curves. Similarly, poor image contrast and focus sharpness introduces errors, especially to particles whose diameter occupies the entire imaging plane. As a consequence, a slight variation of focus may attribute the particle to either *n* or *n* ± 1 division of the sizing bin, corresponding to a possible error of one size group shift in diameter. Again, such errors may be compensated for only if a greater number of particles are counted. Particle size categorization is aided by careful calibration of the photo-micrographic image pixel using traceable microspheres or micrometre length scales. Particle number density is estimated by dividing the total number of particles counted, by the volume and correcting for the dilution fraction used to prepare the sample.

### Laser diffraction (LD)

In low-angle laser light scattering, also called laser diffraction, the angular distribution and intensity of light scattered from a cloud or dilute ensemble of test particles is measured. If no particles cross the light beam, undiffracted light is focused at the centre of the multi-element photo-detector known as an obscuration detector. In contrast, particles crossing the beam interact with the incident beam through scattering and absorption to generate flux intensities at angles inversely proportional to the size of the individual particles. Simultaneous diffraction on more than one particle results in a superposition of the diffraction patterns of individual particles provided the test particles are moving and diffraction between them is averaged out. The resultant spatial intensity pattern at the detector is then summed and digitized to create an intensity flux pattern. A theoretical model based on the diffraction of particles with particular properties and size distribution, implemented on the analyser, is then fitted to the actual diffraction results. The difference between the measured diffraction pattern and the theoretical diffraction pattern is the portion of the measurement inexplicable by the model. Minimizing this residual minimises the analytical uncertainty. Fraunhofer and Mie scattering theories (see Agrawal 1991; de Boer et al. 1987) are models commonly employed for the *prediction* of LD particle sizing. The Mie scattering model is in general more accurate but sensitive to inaccuracies in the required input parameters of imaginary and real component of the refractive indices. In contrast, the Fraunhofer model has no input requirements but not recommended for particles <25 μm in diameter. While LD remains a popular sizing method largely due to its ease of use, wide dynamic range and speed of measurement, there is concern about the accuracy of its sizing outputs in particular where the Mie-scattering model is employed. For example, since the imaginary and real component of the refractive indices are not always accurately known, Mie-based LD sizing analyses can be rendered inaccurate. Nonspherical and nonhomogeneous particles can return incorrect and outlandish results. Because narrow side-by-side peak separations are of the order 15% to 20%, high resolution measurements are not normally achievable. The need for accurate optical parameters and particle shape means that mixtures of particles with different optical properties and shape cannot be accurately sized. Finally, strongly absorbing particles can present problems as they may not produce a useable scattering signal.

## Materials and Methods

### Materials

Four microsphere suspensions with certified mean diameter and number concentrations traceable to the National Institute of Standards and Technology (Table 1) were supplied by Coulter Electronics Limited (Bedfordshire, UK) and employed as calibration standards. The microbubble ultrasound contrast agent used was SonoVue™ (Bracco SpA) with a reported mean diameter of 2.5 μm and number concentration of 1–5 × 10^8^ microbubbles/mL (Schneider 1999). ISOTON^®^ II (Coulter Electronics Ltd.) was employed as a dispersant and used throughout for the preparation of test samples at the appropriate dilution fractions. Prior to use, ISOTON II was pre-filtered using a 0.45 μm cut-off filter, and gas-stabilised by autoclaving then allowed to stand at the experimental temperature of 21 ± 0.5°C for a few days. ISOTON^®^ II is a phosphate buffer saline comprising 7.9 g/L of NaCl, 70.4 g/L of Na_2_EDTA, 0.4 g/L of KCl, 0.2 g/L of NaH_2_PO_4_, 0.3 g/L of NaF, 0.2 g/L of Na_2_HPO_4_ in water.

### Instrument calibration and optimizations

Unless otherwise stated all instruments were calibrated in accordance with the manufacturer’s recommendation.

The Coulter Multisizer IIe used in this study was fitted with Coulter Z2 AccuComp™ for Windows software version 3.01a (Beckman Coulter Electronics, High Wycombe, UK). Particles were identified and classified by simultaneous two-dimensional (2-D) analysis using volume and conductivity. Volume, as measured by direct current was used to identify the size of the particles. A precision aperture with a diameter of 30 μm was employed. The sample siphon volume was fixed at 50 μL. The aperture current was set to 800 μA and generated a calibration factor, K*d* of 338, allowing a nominal measuring range spanning from approximately 0.6 to 18 μm in diameter. Measurements were conducted using a 16 second time-resolution. All measurement outputs were subsequently validated using the volume mode as a check on measurement reproducibility. Data analyses were performed in 256 logarithmically spaced size channels, giving the number of counts per channel.

The OM set-up used was similar to that described earlier (Sennoga 2010). Briefly, the optical microscopic imager used was built around a Nikon Eclipse 50i (Nikon Instruments Limited, Kingston-upon-Thames, UK) upright microscope. The system comprised a Reichert Bright-Line haemocytometer/counting chamber (Hausser Scientific, Horsham, PA, USA) into which, aqueous suspensions of the test specimen were loaded. The microscope was equipped with an overhead long-working distance objective (×40 magnification; *NA* = 0.75 and returned a resolution of 0.45 μm assuming a wavelength of 544 nm). Magnifications greater than ×40 were not feasible owing to the consequent reduction in optical sampling area as magnification is increased. To capture and store images of the test specimen, the microscope was equipped with a Nikon DXM1200C digital video and stills cooled CCD camera (Nikon Instruments Limited) interfaced to a networked computer. Images with suitable focus and contrast were captured and stored in uncompressed 24 bit TIFF format and subsequently analysed to determine both size and counting estimates using in-house segmentation algorithms implemented in MATLAB (The MathWorks, Natick, MA, USA) (Sennoga 2010). Briefly, the program locates particles in an image, using a simple binary segmentation threshold. It then draws the closed contour of each particle; this is then used to generate a list of particle sizes found in the image. A list of the diameters of all the particles is generated and placed in a Microsoft Excel file. The particles are sorted in ascending order by size, and for each size, the cumulative volume calculated at each level. Calibration was performed by capturing an image of the precision graticule built into the haemocytometer and using this to correlate sample volume to our 2-D images.

The laser diffraction analyser used was a Mastersizer™ 2000 (Malvern Instruments, Worcestershire, UK) equipped with Malvern’s MSX1 small volume dispersion unit. The instrument utilised two light sources: a red laser (λ = 633 nm) supplemented with blue light (λ = 466 nm) from a solid state source. Mie-scattering theory was used to convert light scattering data to particle size distribution. The refractive index of the calibration microspheres was provided by the manufacturer (see Table 1). The refractive index of SonoVue™ was determined experimentally on a polarising microscope using the Becke line method (Kerr 1977; Rutley 1998). To minimise multiple scattering at high particle densities, measurements were conducted at concentrations corresponding to laser light obscuration of ≤15%. Obscuration is described as the fractional loss of light intensity compared with the intensity taken during a background measurement. Diffracted light was measured by a total of 52 sensors and accumulated logarithmically in 77 size bands, spanning a size range of 0.02 to 1000 μm. The experimental protocol used involved taking 1000 readings per second. Each measurement was set to run for 12 s (12,000 readings). The particle size analyses reported in this article are the average of three successive laser diffraction runs, *i.e.*, a total of 36,000 readings. Data were compiled with Malvern’s Mastersizer 2000 software (version 5.4). A general purpose irregular model was used for both the microspheres and SonoVue™ as a regular model ignored particle absorption component. In the irregular model, spherical geometry is assumed, as no theory exists that enables laser light scattering instruments to size particles with a nonspherical shape. This means that the measured scattering distribution arising from nonspherically shaped particles has to be adjusted to account for the irregular light scattering, as such particles are expected to scatter light variably and the experimental to model matching may not be as good for non-spherically shaped particles. Specifically, surface roughness may result in loss of light that is not accounted for by the scattering detectors and these losses may be increased by nonspherical geometry. Thus, trial and error was used to iteratively adjust the imaginary component of the refractive index, by recalculating the Mie fitting with different effective absorption values (0.000–0.010) until the fitting residual calculated by the Malvern Mastersizer 2000 software was <5% arbitrary threshold. Adjustment of the imaginary refractive index to correct for such effects is recommended by ISO document No. 13320-1, which can also be consulted for a discussion of reflections off rough surfaces. Failure to use an effective imaginary refractive index can result in the appearance of a false peak in the size distribution. Although the instrument used is capable of generating a variety of size data, for comparative purposes, we focused on the number-based size frequency distribution (frequency vs. diameter). The mean size diameter generated by LD is based on the volume mean D[4,3] = ∑d^3^/∑d^2^ which, for comparative purposes was subsequently converted to mean diameter, *i.e.*, D[1,0] = sum of all diameters/frequency, using proprietary algorithms provided by the instrument manufacturer. Background signals were measured prior to each test run and were close to baseline in all cases. Particle number concentrations were calculated from the masses of microsphere or microbubbles suspended, divided by the density and volume of dispersant using algorithms supplied by the instrument manufacturer. This value was then multiplied by one hundred to generate a unit-less volume percentage *i.e.*, volume sample/volume dispersant. The measured concentration is generated by the Mastersizer analysis software and reported in the form of a volume percentage.

### Sample preparation and measurement

Test microsphere suspensions were prepared by diluting stock solutions of the microspheres in prefiltered, temperature controlled and gas-stabilised ISOTON^®^ II saline. Prior to dilution, microsphere stock solutions were placed in a shaker (Mini Beadbeater; Biospec Products, Bartlesville, OK, USA) for 5 to 10 min, as an aid in breaking clumps and to enhance uniform distribution. SonoVue™ was prepared according to the manufacturer’s instructions. Briefly, 25 mg of SonoVue™ powder was reconstituted in 5 mL of 0.9% (w/v) sodium chloride solution and the microbubbles activated by shaking. Once activated the microbubble suspension was kept at room temperature (21 ± 0.5°C). Test suspensions of SonoVue™ were prepared immediately prior to determinations of mean diameter and number concentration by suspending an equivalence of 1 mL of SonoVue™ stock solutions in 99 mL of ISOTON^®^ II saline, previously filtered through a 0.45 μm filter and gas equilibrated at 21°C. All stock solutions used were sampled from a single vial of SonoVue™ and used within 3 h of activating the bubbles.

Microspheres with certified mean diameter and number concentration (Table 1) were used to evaluate instrument performance with respect to precision and accuracy prior to assessing the sizing and counting effectiveness of the methods by determining mean diameter and number concentration of SonoVue™ microbubbles.

### Instrument precision and accuracy

Sizing and counting measurements for each of the three techniques were conducted simultaneously *i.e.*, side-by-side at room temperature (21 ± 0.5°C), with samples taken from the same batch. Instrument precision was determined using L3 microspheres with certified mean diameter of 3 μm in diameter and number concentration of 7.5 × 10^6^ microspheres per mL. The precision mean diameter and number concentration values quoted are averages of replicate (*n* = 6) determinations from a single batch of microspheres. Sizing and counting accuracy of each method was validated by determining in triplicate mean diameter and number concentration of L2, L3, L4 and L10 microspheres.

### Assessment of method efficacy using SonoVue™ microbubbles

All measurements were replicated (*n* = 3) to calculate the intra-method variability of each sizing and counting method. Determinations of microbubble size and number concentrations were simultaneously conducted at 21 ± 0.5°C.

### Statistical analyses

Sizing and counting data are average values *i.e.*, mean ± standard deviation. Precision was calculated as pooled standard deviation for each parameter. Statistical analysis was performed on the measured data using Microsoft Excel (Version 2007; Microsoft, Redmond, WA, USA) to determine method accuracy as well as intra-method precision. The difference *i.e.*, lack of agreement between results from each method was calculated for mean diameter and number concentration. The significance of the average difference was tested with a paired two-tailed Student’s *t*-test. Differences were considered significant where the calculated probability *p* < 0.05. The standard deviation for each measurement was calculated by taking the square root of the sum of the squared differences from the mean, divided by the degrees of freedom. The coefficient of variation for intra-method determinations, both for mean diameter and number concentration was calculated using:

$$\mathrm{Intra-method variation=}\frac{\mathrm{Mean}\left[\mathrm{std}\left(\mathrm{Run 1}\right)\mathrm{;std}\left(\mathrm{Run 2}\right)\mathrm{;std}\left(\mathrm{Run 3}\right)\right]}{\mathrm{Mean}\left[\mathrm{Mean}\left(\mathrm{Run 1}\right)\mathrm{;Mean}\left(\mathrm{Run 2}\right)\mathrm{;Mean}\left(\mathrm{Run 3}\right)\right]}$$

where 1, 2 and 3 represent either the number concentration or mean diameter obtained by each method over the replicate runs and std denotes the standard deviation.

## Results

### Instrument precision and accuracy

Table 2 shows the average values (mean ± standard deviation) of replicate (*n* = 6) determinations of mean diameter and number concentration as well as the statistical analysis of intra-method variations of the standardised L3 microspheres by ES, OM and LD. Although the mean diameter and number concentration variations for L3 microspheres only are presented here, it should be noted that values for the other microspheres (see Table 1) based on a lower replicate frequency (data not included) were found to agree with those reported here.

**Fig.1:**
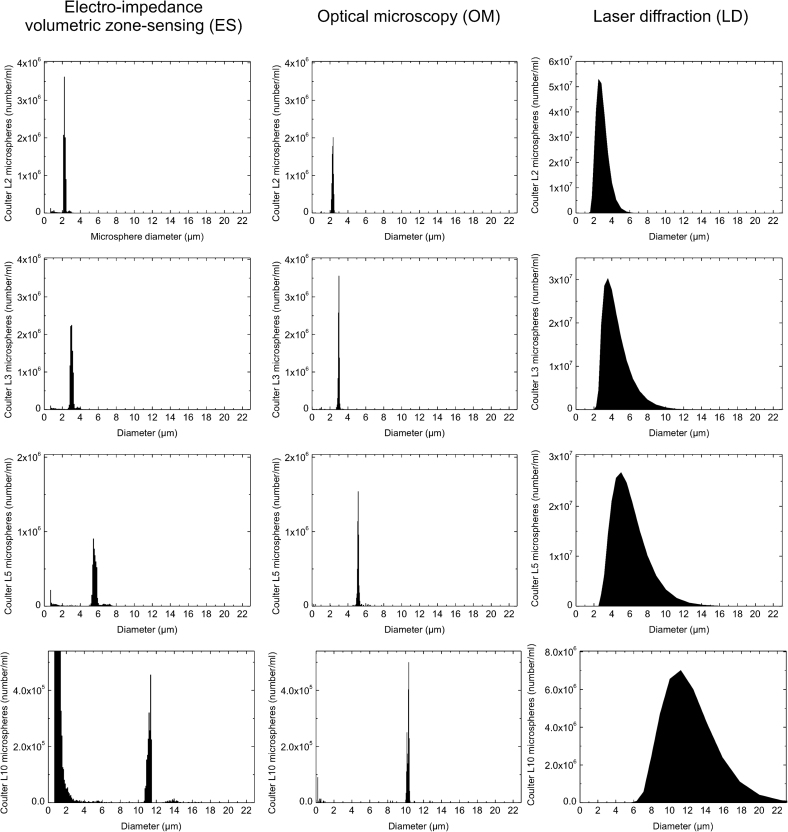
Cumulative microsphere frequency on a number basis plotted against diameter of standardised mono-disperse microspheres (L2, L3, L5 and L10) with certified mean size diameter as measured by electro-impedance volumetric zone sensing (ES), optical microscopy (OM) and laser diffraction (LD). Each distribution is the average of three analyses.

Tables 3 and 4 show, respectively, certified mean diameter and number concentration as well as corresponding ES, OM and LD determinations (expressed as a percentage of certified value) for L3, L4, L5 and L10 microspheres. Figure 1 shows representative size distributions for L3, L4, L5 and L10 microspheres as measured by ES, OM and LD expressed as the cumulative particle frequency on a number basis plotted against mean diameter. ES and OM size distributions for the L2, L3 and L5 microspheres *i.e.*, ≤5 μm in diameters, were characterised by narrow size distributions, which were consistent with the variation associated with the certified values (see Table 1). In contrast, size distributions obtained for the L10 microspheres using ES and OM exhibited an additional peak at the lower end of the size distribution, which is inconsistent with the certified size distribution. For OM, this additional peak is narrow and comparatively small in size compared with that centred at the 10 μm diameter. For ES, this additional peak is broad and larger than that around the expected size of 10 μm. Significantly, the coefficient of variance associated with all L10 measurements using ES was consistently high (CV = 156%) and would under normal circumstances be rejected. In this study, therefore, we have masked the large peak at the lower size range to obtain values with agreeable CV. Indeed this is the value recorded in Tables 4 and 5.

Although, the mean diameter obtained using LD are in good general agreement with the certified, all LD size distributions are characterised by broad size distributions. Peak broadness for the LD number size distributions increased as the size of the microspheres tested increased.

**Fig. 2:**
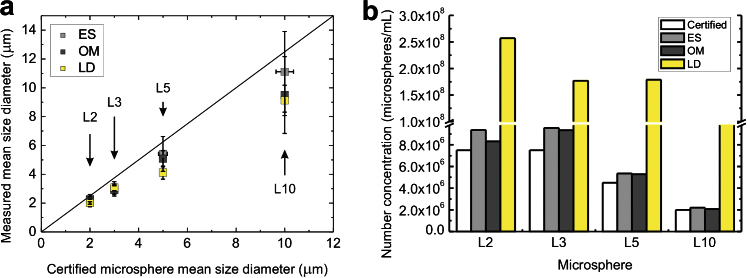
Comparison of experimentally measured mean size diameter (a) and number concentration (b) of standardised mono-disperse L2, L3, L5 and L10 microspheres against certified values.

Figure 2a shows comparative plots of the same experimentally determined mean diameter data plotted against the expected values (*x*-axis). There was general agreement between the experimentally determined mean diameters when compared with certified mean diameter values for microspheres ≤5μm. There is departure from this agreement for the L10 microspheres. It is important to note, however, that since the mean diameter of the calibration microspheres determined increases linearly, perfect agreement between the experimental and expected values is confirmed, when the points in Figure 2a lie on the line y = x.

Figure 2b shows the comparative plots of the certified number concentration plotted side-by-side with the experimental determinations of number concentration. The chart shows general agreement between the certified and experimental number concentration using ES and OM. In comparison, there were significant differences (*p* < 0.05) between determinations of number concentration using LD, compared with the certified.

### Validation of ES, OM and LD microbubble sizing and counting using SonoVue™

Table 5 shows the average values (mean ± standard deviation) of replicate (*n* = 3) determinations of SonoVue™ mean diameter and number concentration. SonoVue™ mean diameter determinations (Table 5) showed submicron repeatability between samples with precision of ±0.06, ±0.01 and ±0.03 for ES, OM and LD, respectively. Table 6 shows the statistical analysis of intra-method variability for SonoVue™ mean diameter and number concentration as determined by ES, OM and LD.

**Fig. 3:**
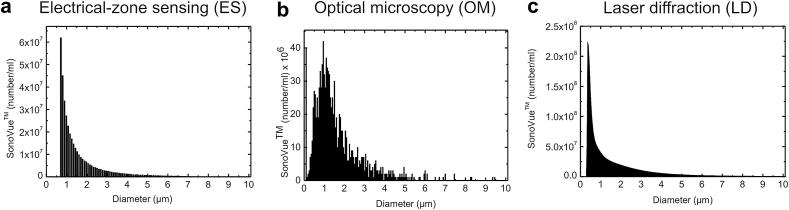
Representative side-by side comparison of SonoVue™ number-weighted size distributions, as determined by (a) electro-impedance volumetric zone sensing (ES), (b) optical microscopy (OM) and (c) laser diffraction (LD).

Figure 3 shows the SonoVue™ number-weighted size-distribution, as measured by (a) ES, (b) OM and (c) LD.

**Fig. 4:**
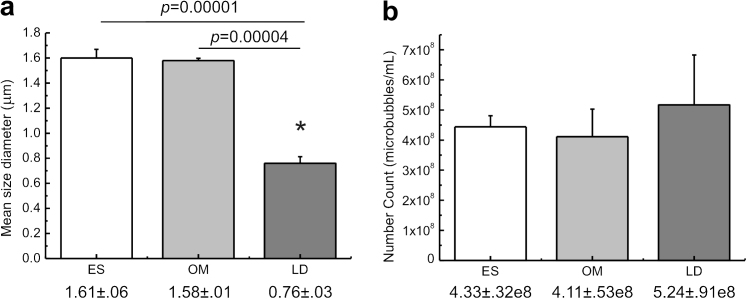
Statistical comparison of (a) experimentally measured mean diameter, and (b) number concentration of SonoVue™ as determined by electro-impedance volumetric zone sensing (ES), optical microscopy (OM) and laser diffraction (LD).

Figure 4 shows bar chart representations of the averaged (*n* = 3) mean diameter and number concentration for SonoVue™ by ES, OM and LD. Statistical evaluation of the analyses showed that there was no significant difference (*p* > 0.05) between the mean diameter obtained using ES (1.61 ± .06 μm) and OM (1.58 ± .01 μm). There were, however, significant differences between the mean diameter obtained using LD (0.76 ± .03 μm) and OM (*p* = 0.00001) as well as between LD and ES (*p* = 0.00004). In addition, there was no significant difference between SonoVue™ number concentration obtained using LD and ES (*p* = 0.247); LD and OM (*p* = 0.192) as well as between ES and OM (*p* = 0.298).

## Discussion

During microbubble sizing and or counting using ES and LD, the bubbles under analysis are subjected, on average to differing rheologic processes. Microbubbles are exposed to intermediate levels of shear stresses as they traverse the sensing zone in ES; or circulate through the analysis cells in LD. In contrast, there is a complete absence of shear stresses in the case of OM because the bubbles are merely loaded in a counting chamber for image capture. In addition, variations in temperature can affect the gas concentration *i.e.,* the partial pressure of gases dissolved in the microbubble dispersing media and lead to a destabilization of the microbubble size (*e.g.*, Mulvana et al. 2010). Whilst the size distributions reported in the literature appear consistent with expectations (Gorce et al. 2000; Greis 2004; Schneider 1999), the impact of these shear stresses on the mean size and number concentration of microbubble dispersions leading to a comparative examination of their sizing and counting efficiencies as well as ascertaining the method with the most reliable outputs, has remained unexplored.

In this article, we first determined the precision of the instruments employed and evaluated particle sizing and counting accuracies for the ES, OM and LD methods using latex microspheres with certified mean diameter and number concentration. Validation of method efficacy was assessed using SonoVue™. Our statistical analyses of replicate ES, OM and LD SonoVue™ mean diameter outputs revealed low (submicron) microbubble sizing variations of ±0.06 μm (4.3% for ES), ±0.01 μm (1.1% for OM) and ±0.03 μm (7.1% for LD). These observations suggest that microbubble sizing is highly reproducible for all three methods when performed by experienced operators. Intra-method variations for SonoVue™ number concentration was low for ES = 8.3% but comparatively high for OM = 22.4% and LD = 32.0%. It is, however, curious to note that the intra-method variation obtained for OM although relatively high is in excellent agreement with the intra-observer variation reported for the same method in our previous study (*i.e.*, 22.4% vs. 23.3% in Sennoga et al. 2010) although the two analyses were conducted by different observers. This observation, we believe offers additional confidence in our experimental execution. The relatively high variation in number concentration recorded for OM is probably due to limitations in the acquisition of the full sample size distribution and arise (see also Sennoga et al. 2010) because: (1) the optical resolution of the system is limited to 0.45 μm and likely excludes bubbles smaller than the 0.45 μm diameter cut-off; and (2) the inability of detecting a large number of bubbles in a reasonable number of micrographic frames. The latter is determined by the original concentration of the suspension. For LD, the variation is probably due to inadequacies in the mathematical description of the scattering flux density leading to poor fitting of the LD data as described in the Description of Techniques section, above.

Direct comparisons of the microbubble sizing and counting outputs showed that there was no significant difference between the number of concentration obtained for SonoVue™ when using either the ES, OM and LD methods (*p* > 0.05). There were, however, significant differences between the mean diameter obtained with LD compared with both ES (*p* = 0.00001) and OM (*p* = 0.00004). These differences in mean diameter suggest that LD under-sized the bubbles. Since the particle size limit detectable by all three methods differs, *i.e.*, 0.6 μm for a 30 μm ES aperture tube (Allen 1990); 0.45 μm for OM (Sennoga et al. 2010) and 0.2 μm (Malvern Instruments, Worcestershire, UK, sales literature) for the Mastersizer 2000 instrument used in this study, it follows that LD is capable of probing microbubbles much smaller than those afforded by ES and OM. It is not surprising, therefore, that a significantly lower mean diameter of 0.76 ± .03 μm was recorded for SonoVue™ in the case of LD compared with mean diameters of 1.61 ± .06 μm for ES (*p* = 0.00001) and 1.58 ± .01 μm for OM (*p* = 0.00004). Analyses of SonoVue™ microbubbles by LD had the effect of shifting the mean diameters to a lower size range. There are several limitations in sizing methodology, which are thought to give rise to differences in mean diameter estimated from the LD, compared with the ES and OM methods. Namely, (1) LD size estimation is inferred from scattering theory and, therefore, highly dependent on model parameters compared with a direct measure of bubble size for OM. Uncertainties in the real and imaginary refractive indices of the bubbles being analyzed, the media used to suspend them, as well as the accuracy of information about particle shape can all influence LD based bubble size estimation; (2) In our study, LD is characterised not only by a larger dynamic range when compared to ES and OM but also a lower detection limit. This suggests that a significant proportion of the particles in the LD size distribution are small bubbles which are not detectable in ES and OM analyses. This interpretation is further supported by, albeit insignificant, but higher bubble counts for the LD analyses as shown in Table 5; and, (3) Unlike OM for which the bubbles under analysis experience no rheologic stress and only minor stresses at the point of measurement in ES, the bubbles in LD analyses are subjected to significant rheologic stresses as they circulate through the measuring cell. Since bubble size is sensitive to gas concentration in the dispersing media *i.e.*, the partial pressure of dissolved gases (see *e.g.*, Mulvana et al. 2010) rapid circulation probably destabilises the dissolved gases in the dispersing media and cause bubbles to shrink or grow in size.

The SonoVue™ number concentrations obtained using all three methods were in general agreement with those previously reported (Schneider 1999). Our evaluation of the ES, OM and LD methods, however, disagreed with previous findings by Sontum (2008). Sontum reported agreement for the mean diameter and number concentration obtained by ES when compared with the alternative methods of OM and LD for the commercial microbubble agent, Sonazoid™ (Daiichi Sankyo Co. Ltd., Tokyo, Japan). It is noteworthy that Sontum’s observations compare well with our ES and OM findings but disagreed in the case of LD. Unfortunately, a comparative examination of our findings with those reported by Sontum is not possible at this stage. This is because Sontum listed neither the comparative ES, OM and LD data nor the associated uncertainties, making it difficult to critically compare both data sets.

In summary, there are several possible explanations for the differences noted between the methods evaluated here. First, although OM allows visualising the microbubbles, the actual number of microbubbles counted is usually small *i.e.*, 10s of thousands compared to 100s of thousands, and accuracy is, therefore, compromised as demonstrated by higher variations of 22.4% for the OM determinations of number concentration compared with the automated ES method (Table 6).

**Fig. 5:**
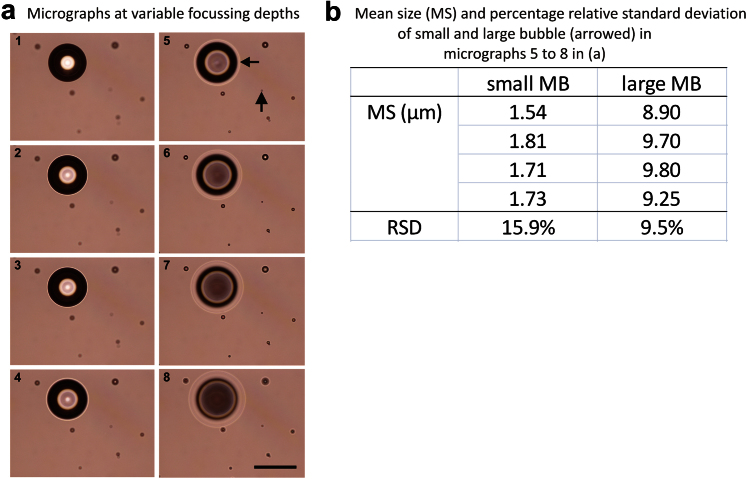
Potential focusing effects in OM sizing of microbubbles, (a) depicts a sequence of optical micrographs (1–8) showing variations of bubble size as the image sampling plane is changed, (b) the sizing analysis of two microbubbles (arrowed) returned size variability (expressed as percentage relative standard deviation) of 9.5% for the large (>7 μm) bubble and 15.9% for the small bubble.

Second, because analyses using OM involves the tabulation of sizes collected via a 2-D projection and not from their total volumes, errors associated with the bubble diameters including focusing and adjustment of image contrast can induce inaccuracies in the calculation of mean diameter, as depicted in Figure 5. Here we first captured sequential images with variable focusing and randomly selected two microbubbles (arrowed) that were then subjected to OM sizing analysis. A microbubble size variation expressed as percentage relative standard deviation of 9.5% for the large bubble and 15.9% for the small bubble was obtained. The higher variation recorded for the small bubble suggests that errors associated with focusing and adjustment of image contrast disproportionately contribute to inaccuracies for small bubbles in comparison to larger ones.

Third, since the microbubble size range detectable by each of the three methods is different and LD is capable of probing particles of a much finer size compared with ES and OM, it is not surprising that the SonoVue™ mean diameter recorded for LD, *i.e*., 0.76 ± 0.03 μm, is in comparison significantly lower than the 1.61 ± 0.06 μm recorded for the ES (*p* = 0.00001) and 1.58 ± 0.01 μm (*p* = 0.00004) for OM. Our LD outputs suggest that a sizeable fraction of the SonoVue™ microbubble dispersion is characterised by particles less than 1.0 μm in diameter. There, however, remains a strong possibility that these submicron particles are in-fact mixed colloidal aggregates such as vesicles, liposomes and micelles of the SonoVue™ amphiphilic components. A pragmatic justification for ignoring bubbles with diameter <1.5 μm can be made on the grounds that these small bubbles contribute negligibly (Gorce et al. 2000) to image contrast enhancement, at least for the ultrasound frequencies of clinical interest. Since our interest in this study focuses on the sizing and counting ability of the methods, we are obliged to consider all particles, regardless of size.

Finally, as pointed out earlier, the accuracy of Mie-based LD sizing and counting analyses is highly dependent on the accuracy of the optical parameters *i.e.*, the real and imaginary refractive indices of the particles being analysed (Beekman et al. 2005), the media used to suspend them, as well as the accuracy of information about particle shape; there remains the possibility that where optical parameters are erroneously set the mean diameter and number concentration determined by LD can be rendered inaccurate.

## Summary and Conclusion

In conclusion, comparison of microbubble mean diameter and number concentration by the ES, OM and LD methods revealed that both ES and OM are highly reproducible methods when conducted by trained observers and returned levels of SonoVue™ mean diameter variations *i.e.*, ES = 4.3%, OM = 1.1%; and variations in SonoVue™ number concentration *i.e.*, ES = 8.3%, OM = 22.4% that were sufficient for our purposes. To our knowledge, this is the first study that has attempted to systematically ascertain the method, which gives the most reproducible measure of the mean diameter and number concentration of gas-filled ultrasound contrast agents. Since the imaginary refractive indices of both the dispersing media and particles can significantly impact the particle size results (see *e.g.*, Beekman et al. 2005) and is not directly measurable, we believe we cannot recommend LD as a suitable method for determining the mean diameter and number concentration of microbubbles. In our opinion, these uncertainties cannot be sufficiently controlled in a routine laboratory analysis. Furthermore, we believe that no single method is fully suited to both sizing and counting of microbubbles. Rather we posit that the best method for determining microbubble mean diameter and number concentration should be governed by the nature of the sample and physical characteristics (size distributions, arithmetic mean size diameter, number concentration, etc) desired. Hypotheses regarding relationships between LD sizing outputs and microbubble sample concentration (obscuration), real and imaginary refractive indices are explored in another publication.

## Figures and Tables

**Table 1:** Certified mean diameter, number concentration and refractive indices of the microspheres and microbubbles used

Identifier	Part No.	Lot No.	Mean diameter (± standard deviation)	Number concentration	Refractive index
			Diameter (μm)	(microspheres/mL)	
L2∗	6602790	931214F	2 (±.134)	7.5 × 10^6^	1.5793
L3∗	6602790	929101F	3 (±.134)	7.5 × 10^6^	1.5793
L5∗	6602790	901202F	5 (±.183)	4.5 × 10^6^	1.5793
L10∗	6602790	974725F	10 (±.360)	2.0 × 10^6^	1.5793
SonoVue™	BR-1	6A029B	2.5†	1–5 × 10^8^†	1.0007‡

∗ Traceable to the National Institute of Standards and Technology.

† See (Schneider 1999).

‡ Determined by Becke line method.

**Table 2:** Experimentally determined and statistical values for intra-method variation of mean size and number concentration of the certified† mono-disperse L3 microspheres as determined by electro-impedance volumetric zone sensing (ES), optical microscopy (OM) and laser diffraction (LD)

Method	Mean diameter		Number concentration	
	∗Mean ± standard deviation (μm)	Intra-method variation (%)	∗Mean ± standard deviation (×10^6^ microspheres/mL)	Intra-method variation (%)
ES	2.99 ± 0.01	0.5	9.55 ± 0.14	1.5
OM	2.98 ± 0.02	0.7	9.35 ± 0.76	11.2
LD	3.05 ± 0.04	1.4	17.7 ± 4.55	12.6

∗ Mean ± standard deviation (n=6).

† Certified mean diameter = 3.0 μm in diameter and Concentration = 7.5 × 10^6^ microspheres/mL $\mathrm{Intra-method variation=}\frac{\mathrm{Mean}\left[\mathrm{std}\left(\mathrm{Run 1}\right)\mathrm{;std}\left(\mathrm{Run 2}\right)\mathrm{;std}\left(\mathrm{Run 3}\right)\right]}{\mathrm{Mean}\left[\mathrm{Mean}\left(\mathrm{Run 1}\right)\mathrm{;Mean}\left(\mathrm{Run 2}\right)\mathrm{;Mean}\left(\mathrm{Run 3}\right)\right]}\mathrm{\times 100}$

**Table 3:** Certified and statistically analysed mean diameters (expressed as a percentage of the certified value) of four different microspheres as determined by electro-impedance volumetric zone sensing (ES), optical microscopy (OM) and laser diffraction (LD)

Microsphere	Certified mean diameter in μm (Uncertainty in %)	Measured mean diameter in % of certified value		
		ES	OM	LD
L2	2 ± 0.134 (6.7)	113 ± 15	114 ± 7	101 ± 13
L3	3 ± 0.134 (4.5)	100 ± 17	97 ± 10	103 ± 7
L5	5 ± 0.183 (3.7)	108 ± 22	102 ± 11	82 ± 11
L10	10 ± 0.360 (3.6)	111 ± 25	95 ± 28	91 ± 11

**Table 4:** Certified and statistically analysed number concentrations (expressed as a percentage of the certified value) of four different microspheres as determined by electro-impedance volumetric zone sensing (ES), optical microscopy (OM) and laser diffraction (LD)

Microsphere	Certified number concentration microbubbles/mL	Measured number concentration in % of certified value		
		ES	OM	LD
L2	7.50E + 06	125	111	3427
L3	7.50E + 06	127	125	2360
L5	4.50E + 06	119	118	3978
L10	2.00E + 06	110	104	1785

**Table 5:** Measured values of SonoVue™ mean diameter and number concentration as determined by electro-impedance volumetric zone sensing (ES), optical microscopy (OM) and laser diffraction (LD)

Method	Mean diameter	Number concentration
	Mean ± standard deviation (μm)	Mean ± standard deviation (×10^8^ number/mL)
ES	1.61 ± 0.06	4.33 ± 0.32
OM	1.58 ± 0.01	4.11 ± 0.53
LD	0.76 ± 0.03	5.24 ± 0.91

Mean ± standard deviation (*n* = 3).

**Table 6:** Statistical values for intra-method variation of SonoVue™ mean diameter and number concentration as determined by electro-impedance volumetric sensing (ES), optical microscopy (OM) and laser diffraction (LD)

Method	Mean diameter	Number concentration
ES	4.3%	8.3%
OM	1.1%	22.4%
LD	7.1%	32.0%
